# Efficacy of Chinese Herbal Medicine as an Adjunctive Therapy on in-Hospital Mortality in Patients with Acute Kidney Injury: A Systematic Review and Meta-Analysis

**DOI:** 10.1155/2016/7592705

**Published:** 2016-04-03

**Authors:** Tuo Chen, Libin Zhan, Zhiwei Fan, Lizhi Bai, Yi Song, Xiaoguang Lu

**Affiliations:** ^1^Emergency Department, Affiliated Zhongshan Hospital of Dalian University, Dalian No. 6, Jiefang Street, Zhongshan District, Dalian, Liaoning 116001, China; ^2^Dalian Medical University, Lvshunkou District, Dalian, Liaoning 116044, China; ^3^College of Basic Medicine, Nanjing University of Chinese Medicine, Nanjing 210000, China

## Abstract

*Objective*. We aimed to systematically assess the efficacy of Chinese herbal medicine (CHM) as an adjunctive therapy on in-hospital mortality in patients with acute kidney injury (AKI).* Methods*. We did a systematic review of articles published in any language up until Jun 23, 2015, by searching PubMed, Embase, the Cochrane Library, CBM, and CNKI. We included all RCTs that compared outcomes of patients with AKI taking CHM plus Western treatment (WT) with those taking WT alone. We applied Cochrane risk-of-bias tool to assess the methodological quality of the included trials.* Results*. Of 832 citations, 15 studies involving 966 patients met inclusion criteria. The methodological quality was assessed with unclear risk of bias. In the primary outcome of meta-analysis, pooled outcome of in-hospital mortality showed that patients randomly assigned to CHM treatment group were associated with low risk of in-hospital mortality compared with those randomly assigned to WT alone (RR = 0.41; 95% CI = 0.24 to 0.71; *P* = 0.001).* Conclusions*. CHM as an adjunctive therapy is associated with a decreased risk of in-hospital mortality compared with WT in patients with AKI. Further studies with high quality and large sample size are needed to verify our conclusions.

## 1. Introduction

Acute kidney injury (AKI) is a clinical syndrome characterised by a rapid reduction in kidney's excretory function with the accumulation of end products of nitrogen metabolism (urea and creatinine) or the loss of urine output. Until now, AKI is a worldwide public health problem associated with substantial morbidity and mortality [1, 2]. Epidemiologic surveys have identified that the estimated number of AKI ranges from more than 5000 cases per million people per year without dialysis-requiring AKI to 295 cases per million people per year with dialysis-requiring disease [3]. Even a small increase in serum creatinine (Scr) level is associated with higher mortality, longer time of hospital stay, and higher cost of care [4, 5].

Prominent progress has been made in the treatment of patients with severe AKI by means of renal replacement therapy (RRT) or continuous renal replacement therapy (CRRT). Nonetheless, mortality remains unacceptably high, with multicentre studies continuing to report rates of more than 30% [6, 7]. Hence, a certain proportion of clinicians have turned to Chinese herbal medicine (CHM) as an adjunctive therapy seeking for an effective treatment by attenuating acute kidney injury and expediting recovery thus improving survival rates.

CHM accelerates kidney recovery and alleviates acute kidney injury caused by blood stasis, fluid stagnation, and qi insufficiency according to Traditional Chinese Medicine (TCM) theory. In recent studies, CHM has shown the comprehensive protection against kidney, heart, brain, intestine, liver, and lung injury in oxidative stress-related disease [8]. The mechanisms for the pharmacological action of CHM for AKI include an antioxidant activity by scavenging reactive oxygen species (ROS) and improving renal levels of superoxide dismutase (SOD) [9, 10] and an anti-inflammatory activity by reducing interleukin-6 (IL-6), interleukin-8 (IL-8), and tumor necrosis factor-alpha (TNF-*α*) levels [11].

Several clinical trials have been published analyzing the beneficial effects of CHM as an adjunctive therapy for AKI. However, there is no critical appraisal of the evidence on whether CHM as a complementary therapy could decrease mortality for patients with AKI. Therefore, we did a systematic review and meta-analysis to provide more reliable evidence on the effect of CHM on survival and other key outcomes.

## 2. Methods

### 2.1. Literature Search and Search Strategy

PRISMA (Preferred Reporting Items for Systematic Reviews and Meta-Analyses) was used to report a systematic review and meta-analysis of trails [12]. Two independent investigators (CT and ZLB) performed a search in PubMed, Embase, the Cochrane Central Register of Controlled Trials, and two Chinese databases including Chinese BioMedical Literature Database (CBM) and China National Knowledge Infrastructure (CNKI) for relevant randomized clinical trials published before June 23, 2015. The following keywords and corresponding titles were used in literature search: “acute kidney injury” or “acute renal failure” or “acute renal injury” or “acute renal insufficiency” or “acute kidney failure” or “acute kidney insufficiency” or “Long Bi” (Characteristic of “Long Bi” presented in Section 4.2) and “traditional herbal medicine” or “traditional Chinese herbs” or “traditional Chinese medicine” or “Chinese herbal drug” or “herbal medicine” or “alternative medicine” or “integrative medicine” and “clinical trails” or “randomized controlled trials”. No restrictions were imposed on publication language and data. We manually searched references and related articles to avoid omissions. We contacted the corresponding authors of the studies for further data if needed.

### 2.2. Study Selection Criteria

Clinical trials regarding the efficacy of CHM as adjuvant therapy for AKI were included if they met the following criteria. (1) Types of participants: patients with AKI were enrolled by authors' criteria. (2) Types of interventions: treatment group participants received both CHM and western treatment (WT) and control group participants received the same WT alone. Other herbal or complementary medicines were not accepted as control group interventions. (3) Types of outcome measures: the primary outcome was in-hospital mortality. The secondary outcome measures included overall efficacy (defined as a three-degree measurement including “cure,” “efficacy,” and “invalid” based on the condition of overall symptom improvement), time of kidney recovery, and adverse events. (4) Types of studies: we included randomized controlled trials (RCTs) concerning CHM plus WT versus WT alone for patients with AKI. Case reports, pharmacokinetic studies, general reviews, animal experiments, and quasi-RCTs where allocation was obtained by use of hospital registry number or date of birth were not considered. Duplicate publications were identified and deleted.

### 2.3. Data Extraction

Two investigators (CT and ZLB) independently extracted the data from eligible trails based on the predefined inclusion criteria. Disagreement between two investigators was resolved by discussion with a third investigator (LXG). Detailed data from including trails were rigorously recorded, which included authors, year of publication, title of study, simple size, age, sex, CHM treatment, WT, treatment duration, in-hospital mortality, overall efficacy, time of kidney recovery, and adverse events. Outcomes reported in 1 or more articles were extracted for meta-analysis.

### 2.4. Risk of Bias Assessment

Study quality was evaluated as recommended in the Cochrane Handbook. Two investigators (CT and ZLB) independently rated each study on the six domains [13]: (1) adequate random sequence generation; (2) allocation concealment; (3) blinding of participants, personnel and outcome assessors; (4) incomplete outcome data; (5) selective outcome reporting; (6) other sources of bias. An assessment of “high,” “unclear,” or “low” risk of bias was provided for each of the above domains. We assessed a trail at high risk of bias when there was one or more domains with high risk of bias. We assessed a trail at low risk of bias when all of domains were with low risk of bias. Otherwise, we assessed a trail at unclear risk of bias. Any disagreement on the risk of bias was resolved by discussion with a third investigator (LXG).

### 2.5. Data Analyses

All statistical analyses were presented by Review Manager 5.3 software (Cochrane Collaboration, Oxford, UK). For dichotomous variables such as in-hospital mortality and overall efficacy, results were presented as risk ratio (RR) with 95% confidence interval (CI). For continuous variables such as time of kidney recovery, we used the mean difference (MD) with 95% CI when outcomes were measured in the same way between trials. We analyzed the heterogeneity by the Cochran *Q* and *I* ^2^ test. There was a considerable level of homogeneity test assessed by using the chi-squared test when the *P* value was less than 0.1. Meanwhile, *I* ^2^ values of 25%, 50%, and 75% correspond to low, medium, and high levels of statistical heterogeneity [14, 15]. We applied a random-effect model when *P* < 0.1 or *I* ^2^ > 50% was considered to indicate a substantial level of heterogeneity [16]. Otherwise, a fixed-effect model was used. Sensitivity analysis was performed by sequentially omitting a single study to assess the potential influence of an individual study. We applied the funnel plot to evaluate publication bias if same outcome measures (i.e., >9) were identified. *P* < 0.05 was considered to be statistically significant.

## 3. Results

### 3.1. Results of Literature Search

**Figure 1:**
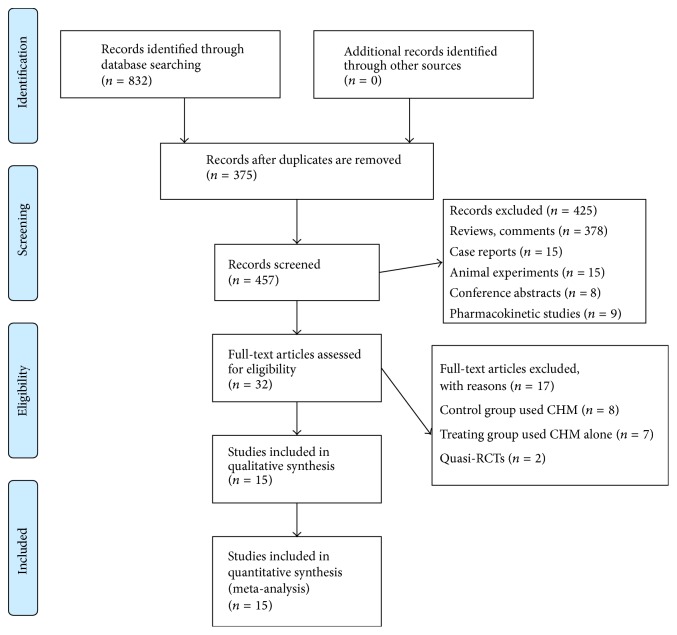
Flowchart of study identification (PRISMA 2009 Flow Diagram). For more information, visit http://www.prisma-statement.org/ [17].

The search process and study selection were depicted in Figure 1. Our search identified 832 potentially relevant citations. After reading titles and abstracts of the articles, 375 studies were excluded because of duplicated publication. We excluded 425 trails due to obviously ineligible inclusion criteria. The remaining 32 trails of full-text papers were analyzed, from which 17 were excluded: 8 because CHM was used in control group, 7 because relevant CHM was applied in treatment group alone, and 2 because patients were allocated by use of hospital registry number. 15 trails were finally retrieved in this systematic review and meta-analysis.

### 3.2. Study Characteristics

15 trails (966 patients) were published in Chinese language and conducted in China between 489 patients randomly assigned to CHM combined with WT in treatment group and 477 patients randomly assigned to WT alone in control group. The included studies were conducted from 2000 to 2014. The sample size in these trails ranged from 30 to 160 participants. All of the trials were parallel arm studies. Participants' gender was reported in 15 trials, of whom 63.25% of participants were male. A variety of data were recorded in these studies, which included in-hospital mortality (12 studies) [18–29], overall efficacy (8 studies) [22–25, 28–31], and time of kidney recovery such as time to oliguria (6 studies) [20–23, 29, 32], time to Scr level recovery (4 studies) [18, 20, 29, 32], and time to Scr and BUN level recovery (4 studies) [21–24]. 12 trails observed treatment duration, ranging from 7 days to 1 month [20–22, 24–32]. Among these studies, all of articles described the component, dosage, and frequency of CHM therapy. The main WT concerning basic treatment, RRT or CRRT, was also demonstrated. All characteristics of the included studies were summarized in Table 1.

### 3.3. Methodological Quality

**Figure 2:**
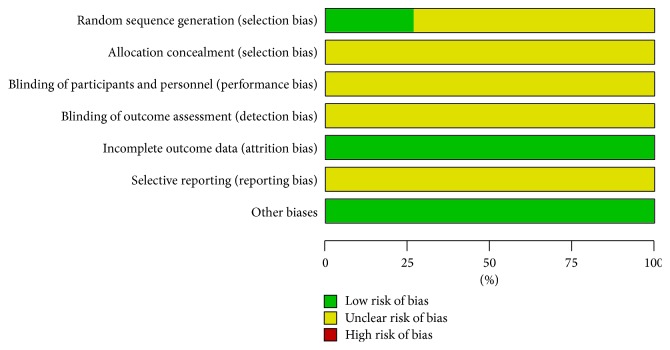
Risk of bias graph.

**Figure 3:**
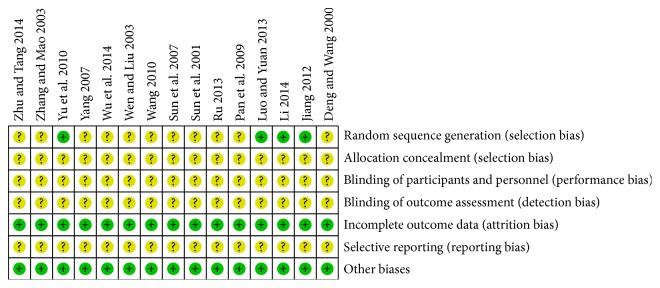
Risk of bias summary. “+”: low risk of bias; “?”: unclear risk of bias.

The methodological quality of all included trails was assessed with unclear risk of bias in Figure 2. 15 included trials were described as randomized controlled trials. Only 4 trails generated adequate randomized sequence by using random number of tables [26, 27, 30, 31]. Among all studies, there was no description on the method of allocation concealment. None of included studies described the double-blind and placebo-controlled method. One trail reported the number and reasons of drop-outs [27] and the other 14 trials reported that all the enrolled patients had completed the trial. All of the 15 trials reported complete clinical outcome data. For other sources of bias, all studies declared no significant difference of enrolled patients at baseline in gender, age, and other pieces of basic information. The details of risk of bias for trails were summarized in Figure 3.

### 3.4. Primary Outcome

#### 3.4.1. In-Hospital Mortality

**Figure 4:**
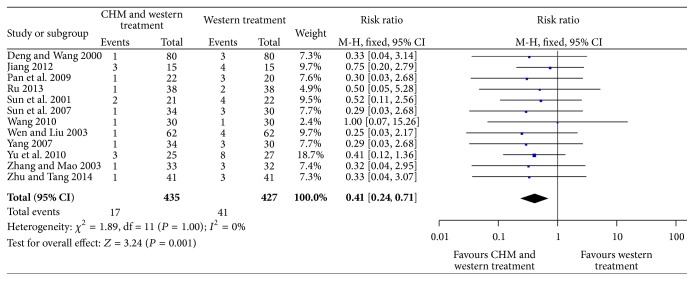
Forest plot for in-hospital mortality.

A total of 12 trails (862 patients) provided data for in-hospital mortality [18–29]. A fixed-effects model was applied to analyze the data due to the low level of homogeneity (*P* = 1.00; *I* ^2^ = 0%). Within 12 trails, as shown in Figure 4, we noted that patients randomly assigned to CHM plus WT had a statistically significant reduction in in-hospital mortality compared with those randomly assigned to WT alone (RR = 0.41; 95% CI = 0.24 to 0.71; *P* = 0.001).

### 3.5. Secondary Outcomes

#### 3.5.1. Overall Efficacy

**Figure 5:**
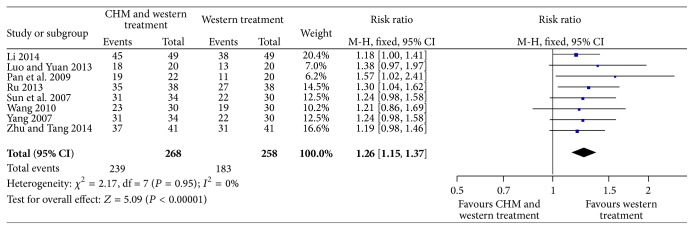
Forest plot for overall efficacy.

A total of 8 trails (526 patients) reported data for overall efficacy [22–25, 28–31]. The data was analyzed using a fixed-effects model based on the heterogeneity test result (*P* = 0.95; *I* ^2^ = 0%). Within 8 trails, as shown in Figure 5, we noted a statistically significant improvement in overall efficacy in patients randomly assigned to CHM as an adjuvant therapy in comparison to those randomly assigned to WT alone (RR = 1.26; 95% CI = 1.15 to 1.37; *P* < 0.00001).

#### 3.5.2. Time of Kidney Recovery

**Figure 6:**
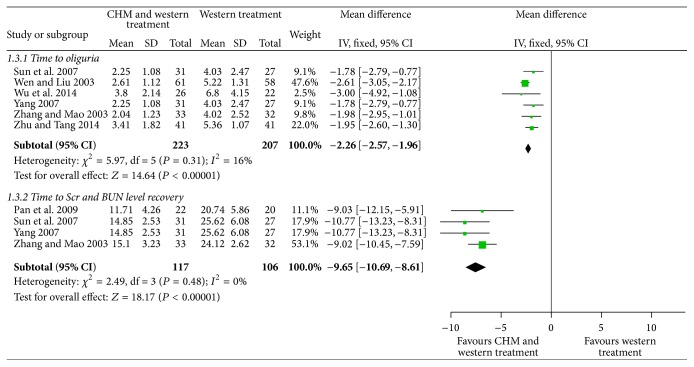
Forest plot for time to oliguria, time to Scr and BUN level recovery.

A total of 6 trails (430 patients) described time to oliguria after treatment [20–23, 29, 32]. We applied a fixed-effects model to analyze the data according to the low level of homogeneity (*P* = 0.31; *I* ^2^ = 16%). Within 6 trails, as shown in Figure 6, we noted that time to oliguria was shortened in patients assigned to CHM as an adjuvant therapy compared with those assigned to WT alone (MD = −2.26; 95% CI = −2.57 to −1.96; *P* < 0.00001).

A total of 4 trails (223 patients) reported on time to Scr and BUN level recovery [21–24]. A fixed-effects model was used to analysis the data based on the low level of homogeneity (*P* = 0.48; *I* ^2^ = 0%). With in 4 trails, as shown in Figure 6, we noted a statistically significant reduction in time to Scr and BUN level recovery in patients taking CHM plus WT compared with WT alone (MD = −9.65; 95% CI = −10.69 to −8.61; *P* < 0.00001).

**Figure 7:**
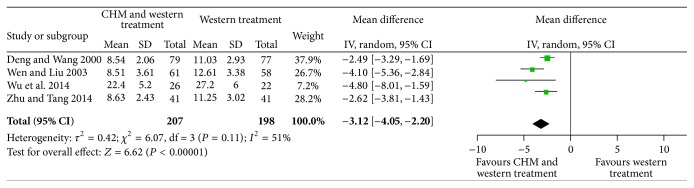
Forest plot for time to Scr level recovery.

A total of 4 trails (405 patients) reported time to Scr level recovery [18, 20, 29, 32]. Based on the medium level of homogeneity (*P* = 0.11; *I* ^2^ = 51%), a random-effects model was applied to analyze the data. Within 4 trails, as shown in Figure 7, we noted that time to Scr level recovery was shortened in patients assigned to CHM combined with WT compared with those assigned to WT alone (MD = −3.12; 95% CI = −4.05 to −2.20; *P* < 0.00001).

#### 3.5.3. Adverse Events

Two trails reported that there were no adverse events [25, 30], while no mention of adverse reactions was reported in the rest of 13 studies. No significant adverse effects were noted in all of included trials.

### 3.6. Sensitivity Analysis

We performed sensitivity analysis testing the robustness of our pooled results by omitting a single study. The sensitivity analysis showed that the summary RRs, MDs with 95% CI for in-hospital mortality (RR range from 0.38 to 0.43), overall efficacy (RR range from 1.24 to 1.28), time to oliguria (MD range from −2.35 to −1.95), time to Scr and BUN level recovery (MD range from −10.36 to −9.40), and time to Scr level recovery (MD range from −3.53 to −2.26) were statistically similar, which suggested that the results of this meta-analysis were robust.

### 3.7. Publication Bias Assessment

**Figure 8:**
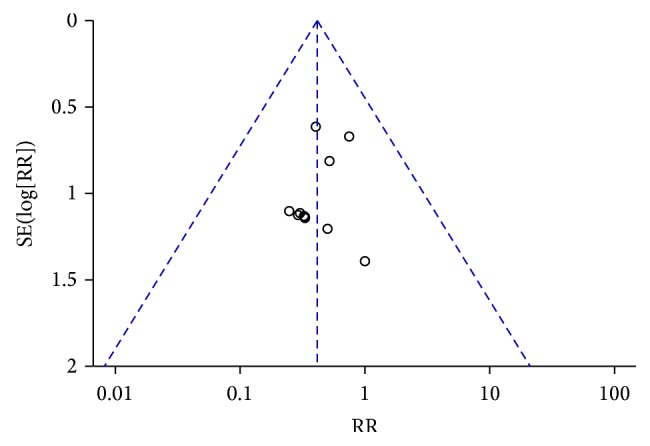
Funnel plot for in-hospital mortality.

In this review, funnel plot suggested possibility of publication bias due to asymmetry (Figure 8).

## 4. Discussion

### 4.1. Summary of Evidences

In the present study, we reviewed 15 randomized controlled trials to evaluate the efficacy of CHM as an adjunctive therapy for patients with AKI. When we analyzed trails, the pooled results of included trials indicated a benefit of CHM as an adjunctive therapy for a significant reduction in in-hospital mortality. There was no clear evidence of a difference in this efficacy by treatment type, scheduling, trial design differences, or patient characteristics. Moreover, the pooled data with AKI has shown that combined therapy significantly improved overall efficacy. On the other hand, we found that the adjunctive use of CHM with WT had significantly shortened time of kidney recovery. These two effects further confirmed the efficacy of CHM as an adjunctive therapy for AKI possibly related to reducing in-hospital mortality.

### 4.2. Strength of Chinese Herbal Medicine for Acute Kidney Injury Treatment

The principles of WT for preventing acute kidney injury are to remove or treat the trigger, to maintain homoeostasis, and to set up extracorporeal renal replacement therapy. After fluid resuscitation and removal of nephrotoxins, CHM as a specific drug-based intervention has been significantly and meaningfully indicated to be nephroprotective. According to Traditional Chinese Medicine (TCM), AKI belongs to the scope of “Long Bi.” In TCM theory, “Long Bi” is caused by blood stasis, fluid stagnation, and qi insufficiency, which is characterised by oliguric syndrome or enuretic syndrome. The principles behind treating these syndromes were through removing blood stasis, reducing fluid retention, and tonifying qi.

In the present study, 5 studies applied the prescription of Xuebijing (XBJ) injection or modified XBJ injection [22–24, 29, 30]. XBJ injection is a representative Chinese patent medicine with the function of removing blood stasis, which is derived from Xuefu Zhuyu decoction recorded in the medical classic Yi Lin Gai Cuo by the Chinese clinician Qingren Wang approximately 200 years ago [33]. Pharmacological studies have indicated that mechanisms of protective effects of XBJ injection against kidney injury may be related to its anti-inflammation effects by decreasing circulating inflammatory cytokines and inhibition of endothelial injury [34, 35]. There were 6 studies which used modified rhubarb decoction (MRD), which had the function of reducing fluid retention [18, 20, 21, 25, 30, 31]. Several experimental researches have shown that MRD or rhubarb extract provides nephroprotection by suppressing the accumulation of end products of nitrogen metabolism, ameliorating tubular epithelial apoptosis, scavenging-free radicals, and augmenting capacity of antioxidant enzymes [36–39]. Astragalus injection or modified astragalus granule in 2 studies has an active function of tonifying qi [19, 26]. In the modern pharmacology research, astragalus provided protection through decreasing the level of malondialdehyde and inhibiting the decline of SOD activity against renal injury [40, 41]. In summary, based on TCM theory and pharmacology research, CHM as an adjunctive therapy for AKI has potentially prominent nephroprotection.

### 4.3. Limitations

Before accepting the above positive findings, the following limitations should also be noted. First, all of included trails were assessed at unclear risk of bias. So many factors that were noted to be unclear may have affected the results. Only 26% (4/15) trails described the details of randomization procedure and allocation concealment was not found in any of the trials. None of included studies described the methods of placebo-controlled and double-blind. None of the studies formally described follow-up and explicitly explained the reasons for the failures at follow-up. Although the authors did not find any related information about adverse effects during the treatment period, it needs more research about whether potential long-term adverse effects are existent in the future.

Second, our funnel plot analysis showed a potential publication bias due to all of included trails published in Chinese, which showed that it is necessary for these types of treatment to be publish in the English resource and to be drawing more attention.

Third, a combination of several herbs was applied in some studies included, and researches about active ingredients of CHM compound in AKI treatment have not been established yet. Therefore, further exploration of the mechanisms of herbal combinations in the treatment of patients with AKI will be required.

Moreover, information about quality control for the development of the herbal manufacture or for the combinations of the herbal products lacks mention, which is one of the obstacles to bring CHM to the world. We hope that more trials should provide complete information about standardization including quality control, compositions, and detailed scheme in the future. Many limitations were existent, whereas we had decreased bias via the process by our methods of studying identification, data selection, statistical analysis, and sensitivity. These efforts should improve reliability and stability of the meta-analysis.

## 5. Conclusion

The preliminary meta-analysis review proved that CHM as an adjunctive therapy for AKI is associated with low risk of in-hospital mortality. As the results of potential publication bias and the methodological drawbacks in the included studies, more rigorously designed, randomized double-blind, placebo-controlled trials with larger numbers of participants are needed to verify our conclusions.

## Figures and Tables

**Table 1:** Characteristics of 15 studies fulfilling the inclusion criteria.

Author (year)	Cases T/C	Age (years) range, mean	Sex M/F	Intervention		Duration	Main outcomes
				Experimental group	Control group		
Deng and Wang 2000 [18]	80/80	NS	T: 59/21, C: 54/26	MRD (100 mL, po, bid) + basic treatment	Basic treatment	NS	In-hospital mortality, time of kidney recovery

Jiang 2012 [27]	15/15	T: 77.4, C: 78.6	T: 10/5, C: 11/4	Jishengshenqiwan pills (90–110 g, po, qd) + basic treatment + CRRT	Basic treatment + CRRT	2 weeks	In-hospital mortality

Li 2014 [31]	49/49	T: 42.2, C: 45.96	T: 28/21, C: 25/24	MRD (50 mL, po, bid) + basic treatment + RRT	Basic treatment + RRT	14 days	Overall efficacy, time of kidney recovery

Luo and Yuan 2013 [30]	20/20	T: 30–53, C: 31–59	T: 13/7, C: 11/9	MRD (200 mL, pr, qd) + basic treatment	Basic treatment	15 days	Overall efficacy

Pan et al. 2009 [24]	22/20	T: 45, C: 47	T: 13/9, C: 12/8	XBJ injection (50 mL, ivgtt, bid) + basic treatment + RRT	Basic treatment + RRT	15 days	In-hospital mortality, overall efficacy, time of kidney recovery

Ru 2013 [28]	38/38	T: 43.83, C: 42.70	T: 22/16, C: 21/17	XBJ injection (50 mL, ivgtt, bid) + basic treatment + RRT	Basic treatment + RRT	2 weeks	In-hospital mortality, overall efficacy

Sun et al. 2001 [19]	21/22	T: 38.6, C: 35.7	T: 14/7, C: 15/7	Huangqi injection (20–30 mL, ivgtt, qd) + basic treatment + RRT	Basic treatment + RRT	2 weeks	In-hospital mortality

Sun et al. 2007 [22]	34/30	T: 38.2, C: 39.1	T: 18/16, C: 17/13	Modified XBJ injection (100 mL, ivgtt, qd) + basic treatment + RRT	Basic treatment + RRT	NS	In-hospital mortality, overall efficacy, time of kidney recovery

Wang 2010 [25]	30/30	T: 54.67, C: 54.7	T: 17/13, C: 18/12	MRD (100 mL, pr, qd) + basic treatment	Basic treatment	1 week	In-hospital mortality, overall efficacy

Wen and Liu 2003 [20]	62/62	T: 17–65, C: 16–63	T: 41/21, C: 39/23	MRD (100 mL, po, qd) + basic treatment + RRT	Basic treatment + RRT	2 weeks	In-hospital mortality, overall efficacy, time of kidney recovery

Wu et al. 2014 [32]	26/22	T: 65, C: 62	T: 20/6, C: 15/7	Dongchongxiacao capsules (2-3 g, po, tid) + basic treatment	Basic treatment	2 weeks	Time of kidney recovery

Yang 2007 [23]	34/30	T: 38.2, C: 42.70	T: 18/16, C: 17/13	XBJ injection (50 mL, ivgtt, bid) + basic treatment + RRT	Basic treatment + RRT	NS	In-hospital mortality, overall efficacy, time of kidney recovery

Yu et al. 2010 [26]	25/27	T: 68.49, C: 69.37	T: 16/9, C: 19/8	Qishen Huoxue granule (10 g, po, tid) + CRRT	CRRT	14 days	In-hospital mortality

Zhang and Mao 2003 [21]	33/32	T: 38.12, C: 37.56	T: 25/8, C: 23/9	MRD (2–4 g, po, tid) + basic treatment + RRT	Basic treatment + RRT	4 weeks	In-hospital mortality, overall efficacy, time of kidney recovery

Zhu and Tang 2014 [29]	41/41	T: 49.2, C: 48.90	T: 28/13, C: 26/15	XBJ injection (50 mL, ivgtt, bid) + basic treatment + RRT	Basic treatment + RRT	7 days	In-hospital mortality, overall efficacy, time of kidney recovery

T: treatment group; C: control group; M: male; F: female; NS: no state; MRD: modified rhubarb decoction; XBJ: Xuebijing; basic treatment: remove the cause + maintain water, electrolyte, and acid-base balance + prevent infection + nutrition support; RRT: renal replacement therapy; CRRT: continuous renal replacement therapy.
